# Physical Properties of Eco-Sustainable Form-Stable Phase Change Materials Included in Mortars Suitable for Buildings Located in Different Continental Regions

**DOI:** 10.3390/ma15072497

**Published:** 2022-03-28

**Authors:** Antonella Sarcinella, José Luís Barroso de Aguiar, Mariaenrica Frigione

**Affiliations:** 1 Department of Innovation Engineering, University of Salento, 73100 Lecce, Italy; antonella.sarcinella@unisalento.it (A.S.); mariaenrica.frigione@unisalento.it (M.F.); 2 Department of Civil Engineering, University of Minho, 4800-058 Guimaraes, Portugal

**Keywords:** Phase Change Materials (PCMs), Thermal Energy Storage (TES), sustainable mortars, Circular Economy (CE), Polyethylene Glycol (PEG)

## Abstract

Starting from two low-cost, low-environmental-impact polymers belonging to the Polyethylene Glycol (PEG) family, i.e., PEG 800 and PEG 1000, two form-stable phase change materials were produced. The two PEGs differ in molecular weight and, as a consequence, the melting and crystallization range of temperatures. The PCMs were obtained, including the PEG, in a liquid state, inside the pores of Lecce Stone flakes, obtained as waste pieces from its processing. A simple and inexpensive impregnation process was selected to produce the PCMs, thus adopting low-environmental-impact materials and cheap processes, and respecting circular economy principles. The two PCMs, the first composed of PEG 800, namely LS/PEG800, and the second composed of a 50/50%wt. mix of the different LS/PEGs, i.e., LS/PEG800_LS/PEG1000, were added as aggregates to four types of mortars, based on aerial and hydraulic lime, gypsum, and cement. The obtained mortars were characterized in their fresh state to assess their workability, and in a solid state after a proper cure to determine their characteristic Latent Heat Thermal Energy Storage (LHTES) properties and mechanical properties in both flexural and compressive modes, taking the mortars not containing any PCM as the reference. The results revealed that, with the proper selection of mortar formulations, it was possible to achieve suitable workability and adequate mechanical characteristics. The selection of a PEG with a low range of phase change temperatures, such as PEG 800, allows one to obtain mortars characterized by a melting/crystallization range that can be considered appropriate in applications characterized by cold climates. The production of a mixed PCM, composed of both PEGs, led to mortars displaying a large interval of melting/crystallization temperatures, which could be suitable in both warm and cold climates.

## 1. Introduction

It is well known that the production of the energy required to regulate the internal temperature of buildings, thus ensuring the optimal comfort of the inhabitants irrespective of the external climate, represents the largest sector of energy consumption. To meet this (ever-growing) global energy demand, large quantities of fossil fuels are needed. Referring to the countries belonging to the European Union, almost 40% of the consumed energy is used for the heating/cooling of buildings [1]; this huge amount is directly related to CO_2_ emissions, causing alarming climate change. The resulting global warming, on the other hand, is rapidly increasing the need for energy to reduce internal temperatures in homes and offices. In this regard, the International Energy Agency (IEA) reports an increase in global energy consumption in the construction sector by 30% in 2060 if the current rate of growth is maintained [2,3,4]. Achieving high levels of energy efficiency in buildings is among the most pressing priorities of governments globally, as this would contribute to reducing energy consumption and the environmental issues associated with CO_2_ emissions. The high concern of the international community toward these issues is proven by the released EU codes and the numerous calls published periodically by the European Commission to support research in this field (such as Green Deal or the new Agenda 2030 for sustainable development goal 7 “Affordable and clean Energy”).

To address this need, Thermal Energy Storage (TES) systems seem to be the most suitable tool because they can store thermal energy and release it later when the reduction in the outdoor temperature can negatively affect the internal comfort of inhabitants [5]. In fact, this role is performed by a phase change material (PCM), which can, in fact, change its physical status according to the environmental temperature. Thus, a PCM can store thermal energy during its phase change from solid to liquid states and release the same thermal energy when it returns to a solid form [6]. In recent years, many different PCMs have been proposed and investigated, and this represents a great advantage since it is possible to select the most appropriate system according to the application in which it must be implemented [7,8]. When the objective to be pursued is the energy efficiency of buildings, the selected PCM can be directly incorporated into mortars [9,10]. Mortars, in fact, can be adapted to many applications, due to their versatility [11]. In addition, they are particularly efficient due to their large heat exchange surfaces [12,13]. So far, according to the literature, the most widely used PCMs are the so-called “microencapsulated PCMs” that can be easily adapted to many different applications [14,15,16]. Lately, a novel way to produce PCMs, i.e., the form-stable method, has been proving to be more advantageous and equally effective.

According to the “form-stable” method, which is widely employed to produce PCMs to include in mortars, the active component of the PCM is absorbed in inert support, which, in turn, will be added as aggregate in the mortar. This procedure is able to prevent polymer leakage, improving the thermal durability of the PCM [17].

In this context, the idea of the project “Development and analysis of mortars, based on different binders, containing original sustainable Phase Change Materials (PCMs) able to improve the energy efficiency of the buildings located in different area” was conceived. The eco-sustainable polymer chosen for the realization of new form-stable PCMs is Polyethylene Glycol (PEG) selected for its favorable characteristics, i.e.,: low toxicity coupled with limited flammability achievable at low costs, all features particularly important in the construction field. Furthermore, PEGs of different molecular weights (MWs) are available. Since MW greatly affects the melting/crystallization temperature range of a polymer, on the basis of the typical outdoor temperatures of a location, it is possible to select the most appropriate PEG, i.e., that possessing a more favorable range of melting/crystallization temperatures to produce the PCM-mortar required in a specific climate condition. As inert support for the form-stable PCM, waste pieces from the processing of a very porous stone are proposed. A local stone has been selected, i.e., Lecce stone, widely employed in many (civil and religious) buildings typical of the Salento region, for the production of friezes and other constructive elements. The utilization of waste materials from other processes, cutting down on costs for their disposal, represents an added value of our choices and respects the circular economy principles. Still in line with the adoption of sustainable and low-cost processes, the inclusion of the polymer in the porous stone granules can be achieved through a simple, cheap procedure, employing a hot plate and a vacuum pump.

PEG polymers, thanks to their adaptability [18], have been proposed to realize different efficient form-stable PCMs [19,20]. They are typically incorporated into porous, organic, or more frequently inorganic, matrices [21,22]. Several studies on the thermal performance of form-stable PEG-based PCMs added to mortars demonstrated that the selection of this polymer leads to materials able to adequately regulate the temperature in a room, thus enhancing the thermal comfort of humans [23,24].

**Figure 1:**
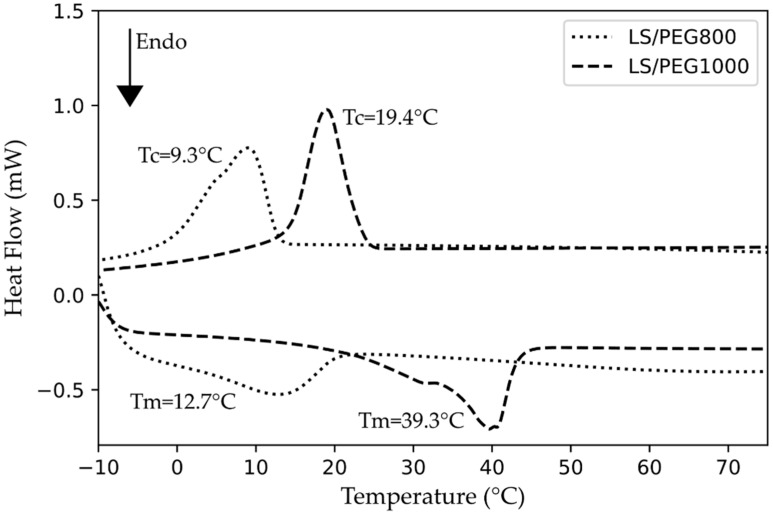
DSC curves of LS/PEG800 and LS/PEG1000 PCM composites, with the indication of the peak temperatures. Data from [25,27].

In our previous studies [25,26], a form-stable PCM based on PEG 1000 was successfully included in mortars produced by different binders. It was found that mortars, composed of hydraulic lime or cement, containing the PEG 1000-based PCM, were able to reduce the energetic heating/cooling needs if applied in buildings located in warm Mediterranean regions [24]. As a continuation of this project, a PEG of a different grade, i.e., PEG 800, was selected on the basis of its melting/crystallization range of temperatures, which shifted towards lower values with respect to PEG 1000. The purpose of this selection was to evaluate whether a PEG800-based PCM, to be included in different mortars, will act as an effective PCM if applied in buildings located in colder climates. In the first paper [27], PEG 800 was thermally characterized, alone or when included in the Lecce Stone granules to form the PCM, in order to assess the thermal characteristics and latent heats. In Figure 1, the calorimetric curves measured by DSC (Differential Scanning Calorimetry) of the PEG-based PCMs produced so far, i.e., LS/PEG800 and LS/PEG1000, are shown.

The DSC data confirmed that, with the selection of a PEG with a lower molecular weight (i.e., PEG 800), it is possible to shift both the melting and crystallization ranges of temperatures of the PEG-based PCM composite to lower values with respect to the LS/PEG1000 PCM. This observation is supported by the peak temperatures of melting and crystallization processes indicated on the curves in Figure 1 for both PEG-based PCMs. The previous observations suggest that LS/PEG800 is a PCM potentially suitable for mortars to be applied in buildings located in cold climates.

The investigation proceeded further with the aim of producing a PEG-based PCM appropriate for a wider range of outdoor temperatures, including both high and low temperatures that can be recorded during the winter and summer seasons, respectively. To address this request, a mixed PEG-based PCM was produced, i.e., a PCM composed of 50 %wt LS/PEG800 and 50 %wt LS/PEG1000.

In this article, the two original PEG-based PCMs, i.e., LS/PEG800 and LS/PEG800_LS/PEG1000, were included in mortars based on different binders, namely aerial and hydraulic lime, cement, and gypsum. The properties of these mortars in fresh and solid states, if compared to the same mortars not containing the two PCMs, were evaluated with the aim to assess if it is possible to produce mortars including these new PCMs, suitable for even demanding applications. The thermal characteristics and latent heats of these mortars were also evaluated with the DSC technique, in order to analyze the phase change temperature intervals and thermal capacity of the mortars and to confirm that the selection of PEG 800 to produce the new PCMs was correct.

## 2. Materials and Methods

### 2.1. Materials

The eco-sustainable, form-stable PCMs were easily and cheaply produced by a combination of a porous stone and a family of low-cost polymers known to display low toxicity and flammability.

**Figure 2:**
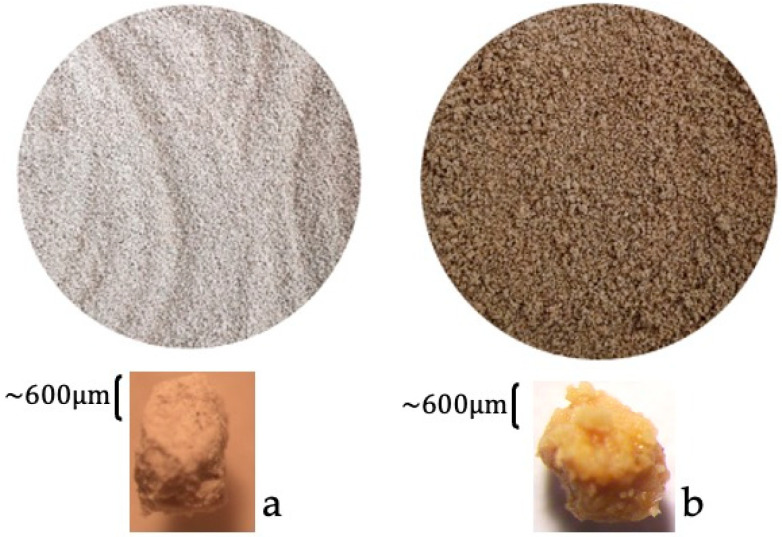
(**a**) Granules of Lecce Stone before the impregnation process; (**b**) granules of Lecce Stone impregnated with PEG800 (i.e., LS/PEG800).

Lecce Stone (LS) is a unique, easily malleable, calcareous limestone extracted in the Salento area (located in the south-east area in Italy). The stone quarries are located close to the city of Lecce, hence the name “Lecce Stone”. During its extraction and workmanship, a large quantity of LS is wasted, posing the problem of its disposal. Starting from the opportunity to valorize a discarded material, waste Lecce Stone fragments were used as the inert support matrix for the form-stable PCMs. LS can, in fact, accomplish this task because it is a very porous material, as our earlier works have demonstrated [25,26]. In particular, characterization of the LS porosity, performed through mercury intrusion porosimetry (MIP), was previously performed and reported in [25]. These measurements revealed that 61% of pore size displays a radius in the range 0.25–2 μm, with an average pore radius of 0.054 ± 0.036 μm, and an open porosity equal to 30.33 ± 0.99%. Flakes of Lecce Stone were sourced from a quarry located in the Salento area (Cursi, Lecce, Italy). The as-received stone fragments were then milled and sieved, and small LS granules were finally obtained (observable in Figure 2a), with a granulometry of 1.6–2.0 mm. The LS particles, impregnated or not with a PEG polymer, were employed as aggregates of the different mortars investigated in the present study.

Two different grades of Poly-Ethylene Glycol (PEG), i.e., PEG 800 and PEG 1000, were selected as the active components of the PCMs. They were both supplied in solid form: PEG 800 was produced by Wuhan Fortuna Chemical Co. (Wuhan, China) and PEG 1000 by Sigma—Aldrich Company (Darmstadt, Germany), respectively.

PEG is a thermoplastic semi-crystalline polymer; it is commercially available in different molecular weights, corresponding to different ranges of melting/crystallization temperatures [18,28]. In a previous phase of the research [25,26,29], a PEG possessing a melting temperature in the range 37–40 °C was selected (i.e., PEG 1000), corresponding to a molecular weight of 1000. Our aim, in that case, was, in fact, the production of a sustainable PCM that could carry out its action, that is to change state from solid to liquid, at the temperatures characteristic of a Mediterranean warm climate; this ability was confirmed by PEG 1000, as illustrated in [24].

Starting with the illustrated results, a PEG with a lower molecular weight, i.e., PEG 800, and a melting range of temperatures around 26–32 °C was analyzed in [27]. The aim was, in fact, the production of a PCM to be added to mortars suitable for cold climates, as in those climates characteristic of countries of Northern Europe. In the continuation of the project, it was established to also manufacture a mixed PCM, composed of both PEG 800 and PEG 1000 polymers. The mixed PCM is expected to be efficient, if included in a mortar, in reducing the indoor temperature fluctuations in a wider range of outdoor temperatures. Therefore, appropriately sized granules of Lecce Stone were impregnated by the PEG 800 polymer, and other LS granules by PEG 1000; in this way, two form-stable PCM composites were obtained, indicated as LS/PEG800 and LS/PEG1000, respectively. LS/PEG800 is visible in Figure 2b. The impregnation procedure, reported in detail in [25], allows one to introduce, under vacuum, the PEGs, brought into the liquid state at appropriate temperatures, into LS granules.

In a previous phase of the research [25,26], the PCM produced with LS/PEG1000 was added to mortar formulations based on different binders (i.e., aerial lime, hydraulic lime, gypsum, and cement) analyzing the properties of the obtained mortars in the fresh state, i.e., their workability, as well as after their curing, determining mechanical properties and latent heat characteristics. As a continuation of the research, the PCMs based on LS/PEG800 and on the mix of LS/PEG800 with LS/PEG1000 (50:50 in weight) were added to mortars based on the same binders (aerial and hydraulic limes, gypsum, and cement mortars), even though greater mortar contents were employed in the present study (1000 kg for 1 m^3^ of each mortar) in order to enhance their mechanical properties. Several physical properties of the produced mortars were assessed. In [27], the characteristics determined on the aerial lime mortar containing LS/PEG800 only were reported. However, for comparison purposes, they are also reported in the present manuscript.

The binders analyzed in this study were supplied by the following: Hydraulic lime (NHL) with a density of 2700 kg/m^3^ was obtained from the CIMPOR company (Lisbon, Portugal); LHOIST (Valverde, Alcanede, Portugal) provided aerial lime, possessing a density of 2450 kg/m^3^; CEM I 42.5 R cement (density of 3030 kg/m^3^) was supplied by SECIL (Lisbon, Portugal); conventional gypsum, possessing high fineness and a density of 2960 kg/m^3^, was finally obtained by SIVAL (Souto da Carpalhosa, Leira, Portugal). All the compositions employed to produce the mortars were based on a binder content of 1000 kg/m^3^, and in order to achieve good mechanical properties in the developed mortars, a superplasticizer (SP) was employed, able to allow a substantial reduction of the mixing water. A superplasticizer, SP, (density of 1050 kg/m^3^) based on polyacrylate commercialized by the BASF company with the trade name Master Glenium SKY 627 (BASF, Porto, Portugal), was employed. The amount of superplasticizer was set at 20 kg/m^3^, in order to appreciably reduce the water content and, thus, improve the (expected) mechanical properties. It has been reported, in fact, that the addition of a PCM to mortars leads to severe reductions in its mechanical characteristics [9,30,31].

For each formulation containing LS/PEG800 or LS/PEG800_LS/PEG1000 PCMs, a reference mortar containing the same amount of non-impregnated Lecce Stone was manufactured for comparison purposes. All the mortar mixtures, of which the compositions are summarized in Table 1, were manufactured according to the European standard EN 998-1 [32].

For each binder, three mortar formulations were manufactured: The first containing the composite material LS/PEG800; another one containing 50%wt. of LS/PEG800 and 50%wt. of LS/PEG1000; and the last, as a control, prepared by introducing pristine LS granules as aggregates.

**Figure 3:**
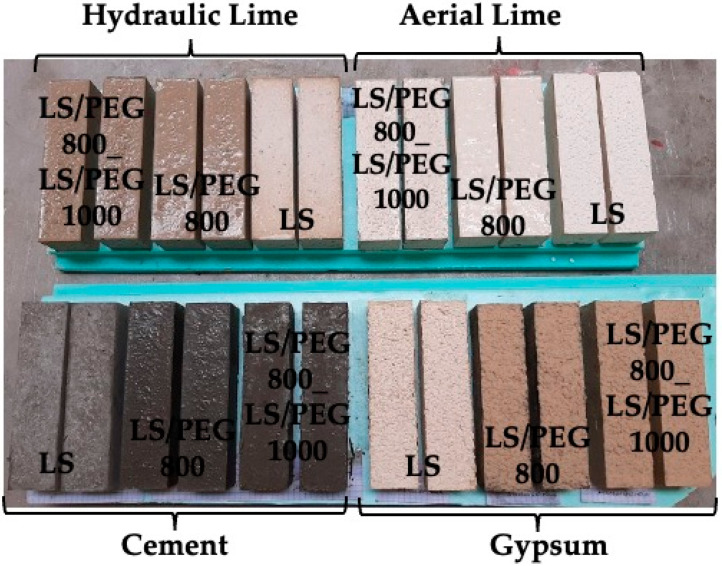
Mortar specimens with indication of the binder and the aggregate used.

A total of twelve compositions were developed, whose specimens can be observed in Figure 3.

### 2.2. Methods

The properties of the manufactured mortars, whose compositions are reported in Table 1, were evaluated in a fresh state (i.e., workability) as well as in a hardened state, determining their thermal properties and mechanical characteristics.

**Figure 4:**
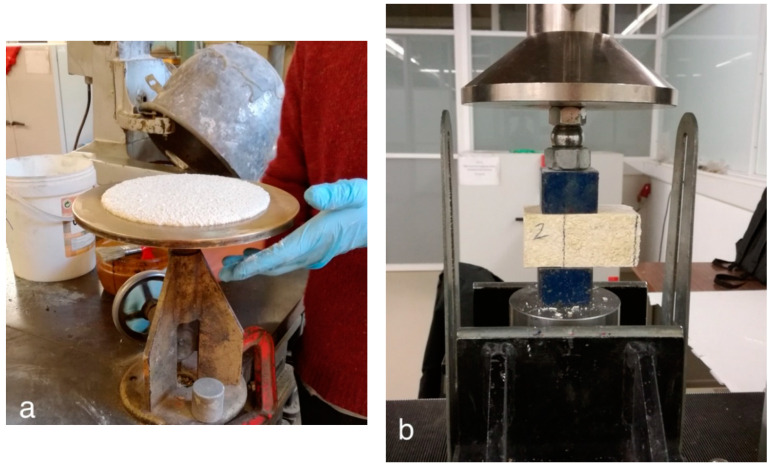
Characterization tests performed on mortars in fresh and hardened states. (**a**) Determination of workability with flow table test; (**b**) measurements of mechanical properties in compressive mode.

The workability test, performed according to the EN 1015-3 standard [33], was performed on fresh mortars employing the flow table method (Figure 4a).

The mortars were then cast in iron molds possessing standard dimensions and cured for 28 days in an environment maintained at standard levels of temperature (25 °C) and humidity (50%).

The mortars, with or without the different PCMs, were thermally characterized with the aid of a Differential Scanning Calorimeter (DSC1 Stare System, Mettler Toledo, Columbus, OH, USA). The melting and crystallization processes of the polymeric phase contained in the PCM-based mortars were analyzed, monitoring the phase change of the LS/PEG composing any PCM. During the DSC analysis, the specimens of the mortars, containing or not one of the two PCMs, were subjected to a heating step from −10 °C to 80 °C and a subsequent cooling stage from 80 °C to −10 °C. The experiments were always carried out under an inert atmosphere employing nitrogen gas with a flow rate of 60 mL/min, and at a heating/cooling rate of 10 °C/min rate was used. This rate was employed in order to compare the calorimetric data found in the present research with those previously measured on both LS/PEG PCMs. Three samples for each mortar (with a weight between 10 and 20 mg) were analyzed, averaging the obtained results.

The mechanical characteristics of the cured mortars were, finally, determined (Figure 4b). The mechanical properties were measured in flexural and compression mode, following the EN 1015-11 standard [34]. For each of the mortar formulations reported in Table 1, 3 prismatic specimens (40 × 40 × 160 mm^3^) were tested. The flexural and compression tests were performed employing a Lloyd dynamometer instrument (LR50K Plus by Ametek Company, Berwyn, PA, USA) at speeds of 6 μm/s and 12 μm/s, respectively.

## 3. Results and Discussion

The physical properties of the mortars reported in Table 1, those containing the two PCM composites (i.e., LS/PEG800 and LS/PEG800_LS/PEG1000) and the control formulations, were analyzed in both fresh and solid states.

The flow table test was first performed on the produced mortars, in order to evaluate their workability and, consequently, their application adequacy. It is well known, in fact, that the workability values of mortar formulations must be comprised in the range of 160–180 mm [35,36]. As it can be observed in Table 2, all the produced mortars show an adequate workability value, confirming the suitability of the selected formulations.

**Figure 5:**
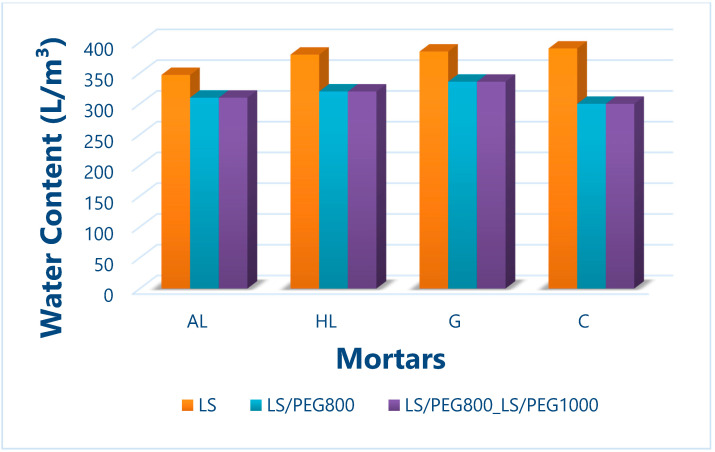
Water content for each mortar composition.

The presence of a high amount of superplasticizer in the fresh mixtures reduced the amount of water required by each mix (as shown in Figure 5), the latter being the prerequisite for obtaining mortars with high mechanical characteristics.

As can be seen in Figure 5, the mortar formulations based on the same binder and containing one of the two PCMs, i.e., LS/PEG800 or LS/PEG800_ LS/PEG1000, were manufactured with the same water content. In this way, it was possible to assess the effect of the employed PCM system on the properties of mortars in the fresh state, as well as in the hardened state. Upon the addition of both PCMs, a decrease in the water content needed to achieve suitable values of workability was recorded with respect to the control mortars. The greatest reduction in water was observed for the mortar based on cement. This behavior could be attributed to small portions of the PEG800 polymer possessing a low viscosity at room temperature [27], with portions lying on the surface of the Lecce Stone grains that can contribute to improving the workability of the mortar mix with replacement of a limited amount of the required water. This feature can, nevertheless, represent an important advantage, potentially counteracting the detrimental effects on mechanical properties brought about by the addition of a PCM in the mortars, as previously reported [26,37,38,39].

The experimental campaign was then focused on determining the properties of the mortars, alone or containing the two PCMs, in the solid state, i.e., after adequate curing. The measurement of the thermal properties of the mortars allowed us to assess if the selection of PEG 800, and the production of two PCMs (i.e., LS/PEG800 and LS/PEG800_LS/PEG1000) based on this polymer, achieved the expected results in terms of suitable melting/crystallization temperature ranges. Determination of the mechanical properties helped to identify the best-performing mortars, i.e., those exhibiting suitable values of compressive and flexural strengths.

**Figure 6:**
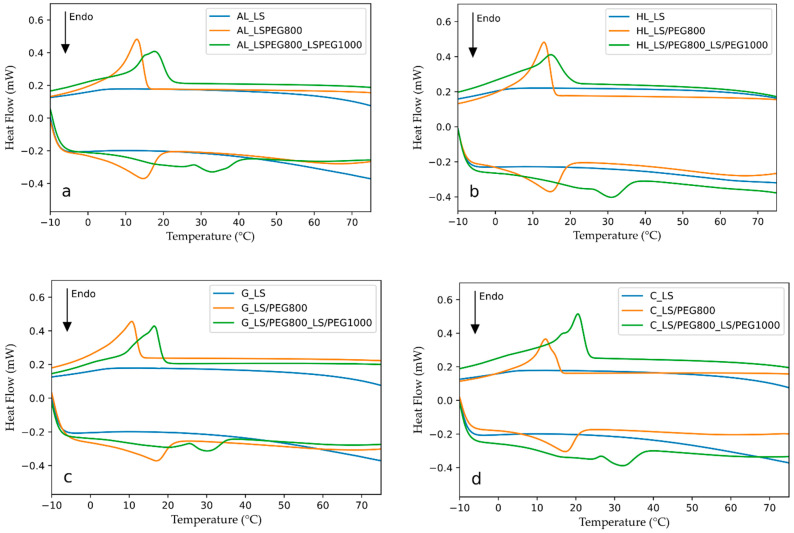
DSC curves recorded for mortars, containing LS/PEG800 and LS/PEG800_ LS/PEG1000 PCM composites or not, based on (**a**) aerial lime; (**b**) hydraulic lime; (**c**) gypsum; (**d**) cement.

In Figure 6, the DSC curves of the mortars produced in this study are presented. For comparison purposes, the thermograms relative to the aerial lime mortar, containing the LS/PEG800 composite or not and previously determined [27], are also reported in Figure 6a. From the analysis of the DSC curves, the values shown in Table 3 were obtained. In particular, the characteristic temperatures (initial, peak, and final temperatures) monitored during the melting and the crystallization processes, and the relative latent heats measured on the produced mortars, containing LS/PEG800 or LS/PEG800_ LS/PEG1000 PCMs or not, are summarized. To facilitate evaluations and comparisons of data, the thermal characteristics previously determined on the mortar AL_LS/PEG800 are shown in Table 3 [27].

Observation of the calorimetric curves reported in Figure 6 allows us to conclude that the mortars not including any PCM based on a polymeric phase do not exhibit any melting/crystallization processes, irrespective of the kind of binder. No peaks are, in fact, noted in the range of temperatures analyzed.

Conversely, peaks related to the melting of the PCM polymeric component in the heating stage and its crystallization in the cooling stage, are always present in the DSC thermograms recorded on the mortars containing a PCM system. Comparing the melting/crystallization initial and final temperatures, as well as the relative peaks, reported in Table 3, the following conclusions can be drawn: The peak temperatures for both melting and crystallization processes depend on the specific PCM added to each mortar; both the melting and crystallization processes are shifted to lower temperatures in the case of mortars containing the LS/PEG800 PCM with respect to those containing the LS/PEG800_ LS/PEG1000 composite. In addition, the melting/crystallization ranges of temperature are larger when the mortars include the LS/PEG800_ LS/PEG1000 PCM; for such mortars, the melting process is completed at approximately 39–45 °C. In the case of mortars including LS/PEG800 PCM, the melting process ends before 26 °C. The initial melting temperature, on the other hand, ranges between −2 and 6 °C, with no clear trend with respect to the kind of mortar or the added PCM; it essentially depends on the initial melting temperature of the polymer possessing the lower melting temperature, in all cases being PEG 800. It can be concluded, therefore, that the kind of mortar has no effect on melting/crystallization ranges of temperature, nor on the peak temperatures. Similar considerations can be made by analyzing the calorimetric data relating to the crystallization process, with the initial crystallization (solidification) temperatures depending on the kind of PCM and the final value being similar for all the mortars, basically depending on the presence of PEG 800 in both PCMs.

The previous observations confirm the hypothesis that led to the selection of the PEG 800 to produce a PCM displaying a phase change range of temperatures shifted to lower values with respect to PEG 1000, thus efficient in colder climates. As already mentioned, in fact, a form-stable PCM composite based on PEG 1000, i.e., LS/PEG1000, was demonstrated to be able to reduce the energetic heating/cooling needs when added to mortars used in buildings located in warm climates [24]. On the other hand, the realization of a PCM based on two different polymers characterized by different molecular weights (i.e., PEG 800 and PEG 1000), thus exhibiting different melting/crystallization ranges, is a good omen for obtaining mortars containing PCMs that are effective at both low and high external temperatures.

As observed in Table 3, the latent heats for melting and crystallization processes for all the mortars containing a PCM range between 8 and 12 J/g. These values appear independent of both the type of mortar and the added PCM. As already underlined, in the range of temperatures analyzed, the thermal characteristics of the mortars containing a PCM depend on the thermal characteristics of the polymeric phase of the specific PCM. It is expected, therefore, that the kind of mortar cannot affect the melting/crystallization enthalpies. Furthermore, since very similar melting/crystallization enthalpies were measured on the two LS/PEG PCMs (LS/PEG800: ΔHm = 28.3 J/g, ΔHc = 28.1 J/g; LS/PEG1000: ΔHm = 27.7 J/g, ΔHc = 26.2 J/g) [27], this explains why the type of composite PCM, i.e., LS/PEG800 or LS/PEG800_ LS/PEG1000, has a negligible influence on the measured latent heats, provided that the amount (% in weight) of the PEG in each PCM composite is the same in the produced mortars, as in the present case (the amount of both PEG 800 and PEG 1000 absorbed in the porous Lecce Stone granules is, in fact, similar, and equal to 23% [25,27]).

The values of melting/crystallization enthalpies found in the present study are roughly in line with those previously reported for mortars containing the LS/PEG1000 PCM, mortars based on the same binders even if in a different composition [26]; it was demonstrated that the latent heats of these latter mortars were sufficient to influence the internal temperature of an environment [24]. This observation suggests, once again, that the mortars containing one of the two PCMs produced in this study, i.e., LS/PEG800 or LS/PEG800_ LS/PEG1000, will also be able to act as an energy storage system when enclosed in mortars applied in buildings.

The mechanical properties (tested in flexural and compressive modes) of the mortar formulations reported in Table 1 were, finally, assessed after 28 days of aging. Table 4 reports the measured values of flexural and compressive strengths recorded for each mortar, with the indication of the classification according to the standard EN 998-1 [32].

As expected, an important decrease in mechanical properties is generally observed as a consequence of the introduction of a PCM, irrespective to the kind of PEG composing the PCM. This behavior has been observed in several other studies; with the inclusion of PCM, the mechanical properties of mortars can significantly decrease [23,38,39]. Several reasons may explain this occurrence: (i) An increase in the porosity of the mortar containing the PCM; (ii) a delay of the hydration reaction of the mortar; and (iii) in the case of a form-stable PCM, as in the present study, the polymeric component that constitutes the PCM, a part of which can be located on the surface of the inert support, can take part in the mixing of the mortar, reducing its mechanical properties.

**Figure 7:**
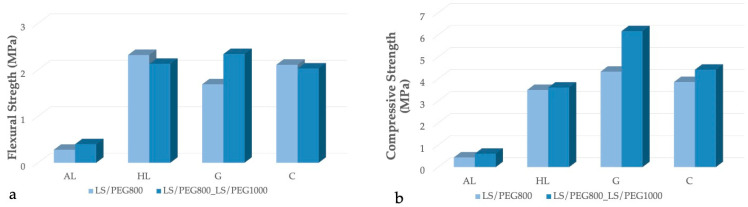
(**a**) Values of flexural strength recorded for the mortars containing the different PEG-based PCMs; (**b**) values of compressive strength recorded for the mortars containing the different PEG-based PCMs.

No specific trend can be attributed to the PCM composition when measuring the flexural strength. Referring to the compressive strength, slightly greater values of this property are achieved when adding the mixed PCM, i.e., that produced with the 50/50 % wt LS/PEG800_LS/PEG1000 composite, particularly in the case of the mortars based on gypsum and cement. To better appreciate the mechanical strength values recorded upon the addition of the two PCMs, the results of the mechanical characterization are additionally illustrated in Figure 7.

Keeping in mind the type of applications in which the PCM-based mortars can be proposed, the compressive strength of the mortars under analysis must at least fall within the CSII class. In this regard, mortars with the experimented PCMs based on hydraulic lime, gypsum, and cement fall within the requirements of the CSII class while those based on aerial lime do not meet the qualification. This result, as already evidenced in our previous work for an aerial lime mortar based on LS/PEG800 [27], was also confirmed in the present study when the LS/PEG800_LS/PEG1000 compound is added to the same binder. It can be concluded, therefore, that the addition of a PCM based on LS/PEG to an aerial lime mortar does not allow it to achieve suitable mechanical characteristics.

The analysis of the flexural and compressive strength data, reported in Figure 7a,b, respectively, for the different mortars containing the experimented PCMs, allows us to conclude that the mortars offering satisfactory (flexural and compressive) mechanical characteristics are those based on hydraulic lime and cement, irrespective of the kind of PCM added. Mortars based on gypsum, on the other hand, displayed a lower value of flexural strength when LS/PEG800 was employed.

Thermal experimental tests are, consequently, in progress on the mortars based on hydraulic lime and cement binders to assess the effective efficiency of LS/PEG-based composites as phase change materials in different climatic environments. The results of these tests, performed in a climatic chamber able to simulate different outdoor climatic conditions while recording the temperature inside a small room covered with the mortar under analysis, will be the subject of a forthcoming manuscript.

## 4. Conclusions

In this work, the main results of the physical analyses conducted on different mortar formulations containing two form-stable PCMs, are presented. The two phase change materials were realized by impregnating very porous Lecce Stone granules with Polyethylene Glycol polymers possessing different molecular weights and, in turn, different melting/crystallization ranges of temperatures, i.e., PEG 800 and PEG 1000. The obtained PCMs, LS/PEG800, and LS/PEG800_LS/PEG1000 were included as aggregates in aerial lime, hydraulic lime, gypsum, and cement mortar mixtures. The effect of the incorporation of each PCM on the workability of the fresh mortar mixtures and on the mechanical and thermal properties of the same cured mortars was evaluated, to establish the suitability and the efficacy of the mortar mixtures produced with the PCMs. Although the addition of both PCMs in mortar led to modifications in their characteristics, in both fresh and solid states, with the proper selection of the formulation, it was possible to obtain mixtures possessing suitable workability and still adequate mechanical properties. In particular, it was possible to obtain mortars, containing one of the PEG-based PCMs, with a compressive strength classified within CSII/CSIII classes, except for the systems based on an aerial lime binder. Referring to the LHTES properties, measured on the mortars with the aid of calorimetric analysis, the selection of a PEG characterized by lower melting/crystallization temperatures allows one to obtain mortars with phase change temperatures characteristic of continental climates, as expected. Furthermore, the mortars containing the mixed PCM, i.e., that composed by LS/PEG800:LS/PEG1000 50:50%wt., showed a large interval of melting/crystallization temperatures, suggesting that this mortar could be suitable in both warm and cold climates.

Thermal experimental tests are in progress in a climatic chamber able to simulate different climatic conditions on some of the best-performing mortars, i.e., those based on hydraulic lime and cement binders, to assess the effective efficiency of LS/PEG-based-composites as phase change materials in different weather conditions.

## Figures and Tables

**Table 1:** Compositions of the produced and analyzed mortars (reported as kg/m^3^ of produced mortar).

Mortars	Binder ^a^/ Content	Aggregates			SP	Water Saturation ^b^	Water	Water/ Binder
		LS	PEG800 Content	PEG1000 Content				
AL_LS	AL/1000	668	0	0	20	168	347	0.35
AL_LS/PEG800		979	225	0	20	0	310	0.31
AL_LS/PEG800_LS/PEG1000		979	113	113	20	0	310	0.31
HL_LS	HL/1000	682	0	0	20	171	380	0.38
HL_LS/PEG800		1082	249	0	20	0	320	0.32
HL_LS/PEG800_LS/PEG1000		1082	124	124	20	0	320	0.32
G_LS	G/1000	763	0	0	20	192	385	0.39
G_LS/PEG800		1129	260	0	20	0	336	0.34
G_LS/PEG800_LS/PEG1000		1129	130	130	20	0	340	0.34
C_LS	C/1000	772	0	0	20	194	390	0.39
C_LS/PEG800		1307	301	0	20	0	300	0.30
C_LS/PEG800_LS/PEG1000		1307	150	150	20	0	300	0.30

^a^ Each binder is indicated with an acronym: AL represents Aerial Lime; HL represents Hydraulic Lime; G represents Gypsum; and C represents Cement. ^b^ The “water saturation” label is the amount of water used to saturate the LS aggregates in order to avoid them to absorb water required to manufacture the mortars. When an LS/PEG composite is used, no additional water was required, since the stone pores were almost completely saturated by PEG 800 or PEG 1000.

**Table 2:** Workability values of the produced mortars (their compositions are reported in Table 1).

Mortars	Workability (mm)
AL_LS	178 ± 2.0
AL_LS/PEG800	160 ± 3.0
AL_LS/PEG800_LS/PEG1000	175 ± 2.0
HL_LS	175 ± 1.0
HL_LS/PEG800	170 ± 3.0
HL_LS/PEG800_LS/PEG1000	163 ± 2.0
G_LS	170 ± 4.0
G_LS/PEG800	165 ± 3.0
G_LS/PEG800_LS/PEG1000	163 ± 1.0
C_LS	180 ± 0.5
C_LS/PEG800	170 ± 1.0
C_LS/PEG800_LS/PEG1000	170 ± 4.0

**Table 3:** Start, peak, and end temperatures and enthalpy measured during melting (heating stage) and subsequent crystallization (cooling stage) on the mortars based on different binders and containing one of the two LS/PEG PCM composites produced in this study.

	System	T_start_ (°C)	Tm (°C)	T_end_ (°C)	ΔHm (J/g)
**Heating Stage**	AL_LS/PEG800	3.3 ± 1.9	15.0 ± 1.0	24.1 ± 1.2	11.8 ± 0.4
	AL_LS/PEG800_LS/PEG1000	4.3 ± 0.9	32.3 ± 0.8	45.2 ± 0.5	9.7 ± 2.1
	HL_LS/PEG800	−2.3 ± 0.8	14.0 ± 0.8	21.2 ± 0.5	9.1 ± 0.9
	HL_LS/PEG800_LS/PEG1000	6.9 ± 3.1	32.4 ± 2.6	38.4 ± 0.8	9.1 ± 1.2
	G_LS/PEG800	2.5 ± 1.1	16.4 ± 0.8	24.6 ± 2.0	7.8 ± 0.6
	G_LS/PEG800_LS/PEG1000	3.2 ± 0.9	30.8 ± 1.6	38.9 ± 2.7	8.1 ± 0.4
	C_LS/PEG800	−2.0 ± 0.8	17.3 ± 0.2	25.8 ± 1.8	9.5 ± 0.5
	C_LS/PEG800_LS/PEG1000	3.5 ± 1.8	33.5 ± 0.2	42.3 ± 0.4	9.7 ± 0.9
	**System**	**T_start_ (°C)**	**Tc (°C)**	**T_end_ (°C)**	**ΔHc (J/g)**
**Cooling Stage**	AL_LS/PEG800	17.7 ± 1.5	13.1 ± 1.1	−6.4 ± 0.9	12.5 ± 1.0
	AL_LS/PEG800_LS/PEG1000	29.0 ± 0.9	18.6 ± 1.6	−0.6 ± 1.2	10.8 ± 1.4
	HL_LS/PEG800	14.6 ± 1.4	12.4 ± 3.0	−3.3 ± 0.6	10.3 ± 1.2
	HL_LS/PEG800_LS/PEG1000	25.1 ± 0.7	15.2 ± 0.5	−1.4 ± 2.2	9.2 ± 3.5
	G_LS/PEG800	15.8 ± 0.2	11.0 ± 0.3	−1.5 ± 0.9	9.2 ± 1.1
	G_LS/PEG800_LS/PEG1000	25.7 ± 1.9	17.5 ± 2.3	−1.8 ± 3.1	9.2 ± 1.8
	C_LS/PEG800	19.1 ± 0.6	13.0 ± 1.3	−0.4 ± 0.2	10.5 ± 1.0
	C_LS/PEG800_LS/PEG1000	25.9 ± 0.5	19.5 ± 2.4	0.3 ± 0.2	11.3 ± 3.4

**Table 4:** Mechanical properties measured in flexural and compressive mode with the classification, according to the standard EN 998-1:2010.

Sample	Flexural Strength (N/mm^2^)	Compressive Strength (N/mm^2^)	Classification EN 998-1:2010
AL_LS	0.63 ± 0.19	1.47 ± 0.16	CSI
AL_LS/PEG800	0.28 ± 0.12	0.44 ± 0.01	
AL_LS/PEG800_LS/PEG1000	0.40 ± 0.01	0.61 ± 0.08	
HL_LS	5.20 ± 1.53	11.66 ± 0.47	CSIV
HL_LS/PEG800	2.32 ± 0.37	3.50 ± 0.15	CSII-CSIII
HL_LS/PEG800_LS/PEG1000	2.13 ± 0.52	3.61 ± 0.54	
G_LS	9.28 ± 1.26	22.34 ± 0.21	CSIV
G_LS/PEG800	1.69 ± 0.04	4.33 ± 1.74	CSII-CSIII
G_LS/PEG800_LS/PEG1000	2.34 ± 0.02	6.17 ± 0.51	
C_LS	11.76 ± 1.11	65.55 ± 6.09	CSIV
C_LS/PEG800	2.11 ± 0.11	3.86 ± 1.21	CSII-CSIII
C_LS/PEG800_LS/PEG1000	2.03 ± 0.18	4.42 ± 0.72	

## Data Availability

The study does not report any data, please exclude the statement.
